# Supplementary material for: Local and Global Variability in Developing Human T-Cell Repertoires
Source: PRX Life. Author manuscript; Available in PMC 2024 Nov 22. (PMC11583800; doi:10.1103/prxlife.2.013011)
Supplement: supplementary figures [file NIHMS1994493-supplement-supplementary_figures.pdf]

Supplementary Information

Variability in the local and global composition of human T-cell receptor repertoires during thymic development across cell types and individuals

Giulio Isacchini, Valentin Quiniou, Pierre Barennes, Vanessa Mhanna, Hélène Vantomme, Paul Stys, Encarnita Mariotti-Ferandiz, David Klatzmann, Aleksandra M. Walczak, Thierry Mora, and Armita Nourmohammad

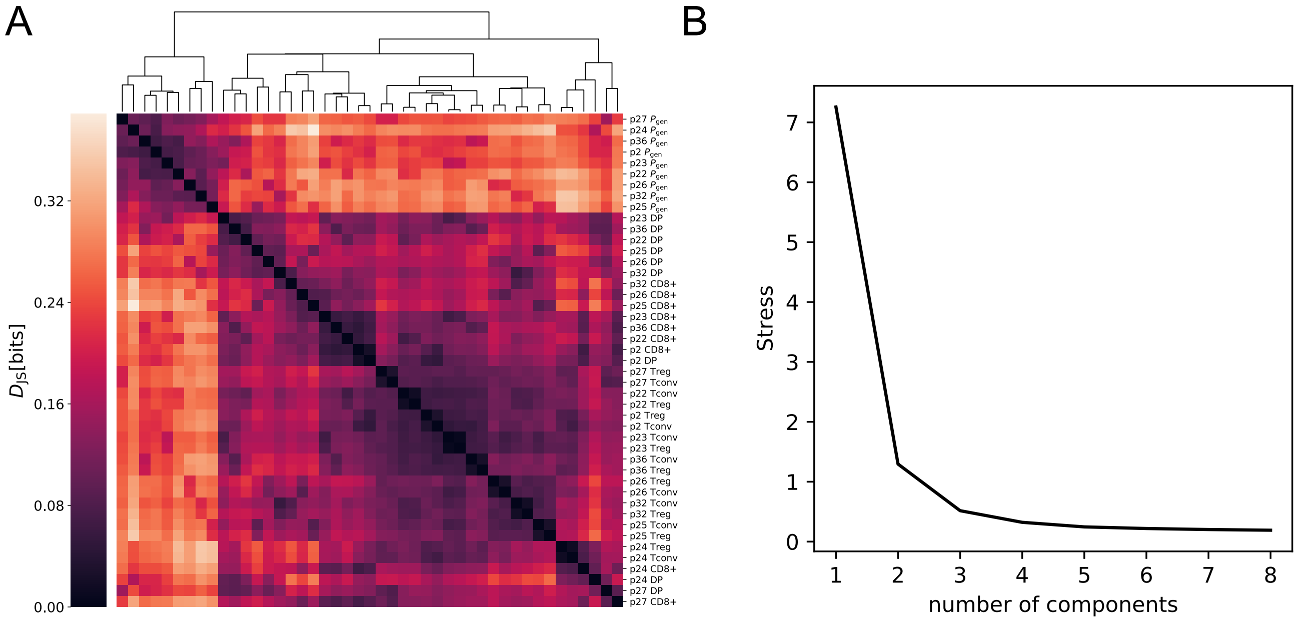

FIG. S1. **(A)** distance matrix $\mathcal{M}_g$ between different datasets. Ordering is performed using the hierarchical clustering algorithm implemented in the seaborn clustermap function. **(B)** Stress as a function of the number of components of the Multi-Dimensional Scaling embedding of the matrix (see Methods).

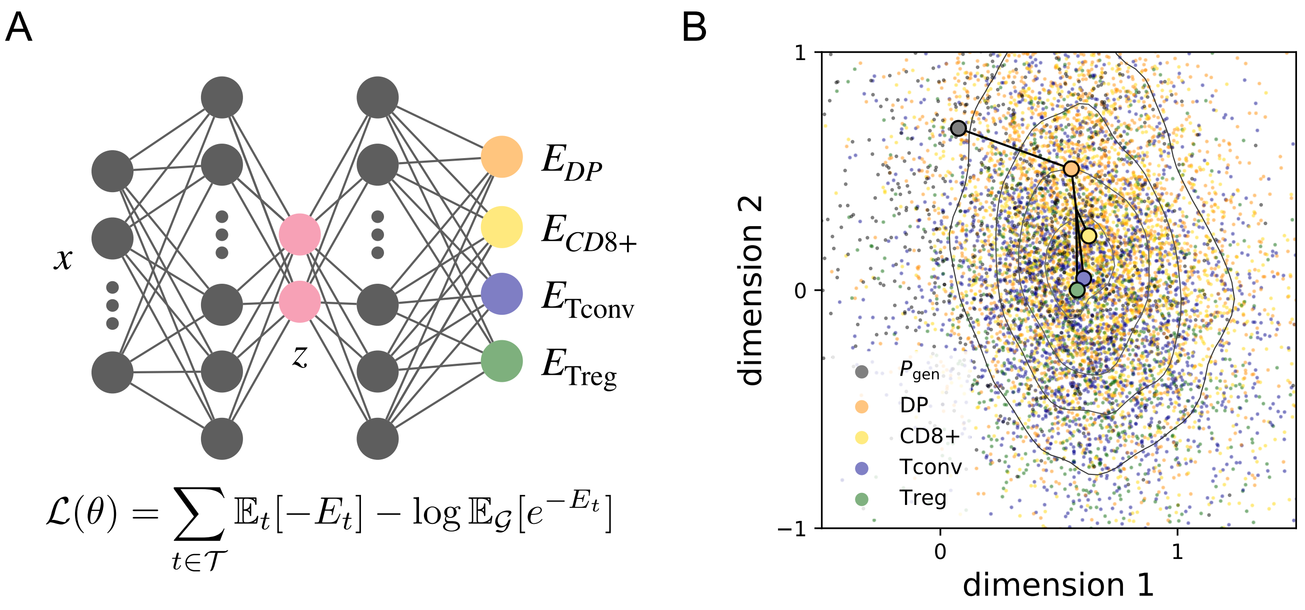

FIG. S2. **(A)** Schematic shows the network architecture for joint inference of selection factors on a shared representation space of the same neural network. **(B)** Vizualization of the two-dimensional representation space z where each point corresponds to a single sequence. No clear separation of subsets is visible in the representation space but the mean value of the distribution follows the differentiation process.

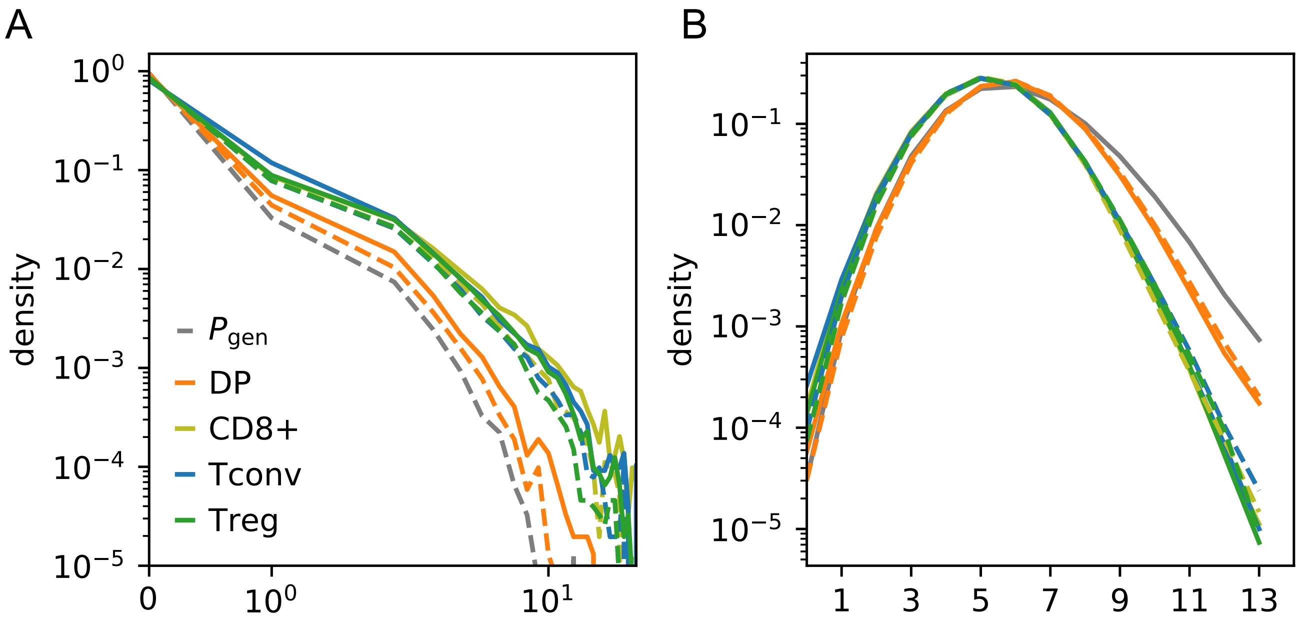

FIG. S3. **Replication of main text Fig 2 with synthetic data. (A)** The probability that a sequence has exactly n nearest neighbours with the same VJ genes is shown. **(B)** The probability that two randomly sampled sequences with the same VJ gene combination have Hamming distance d_H between their aminoacid sequences is shown.

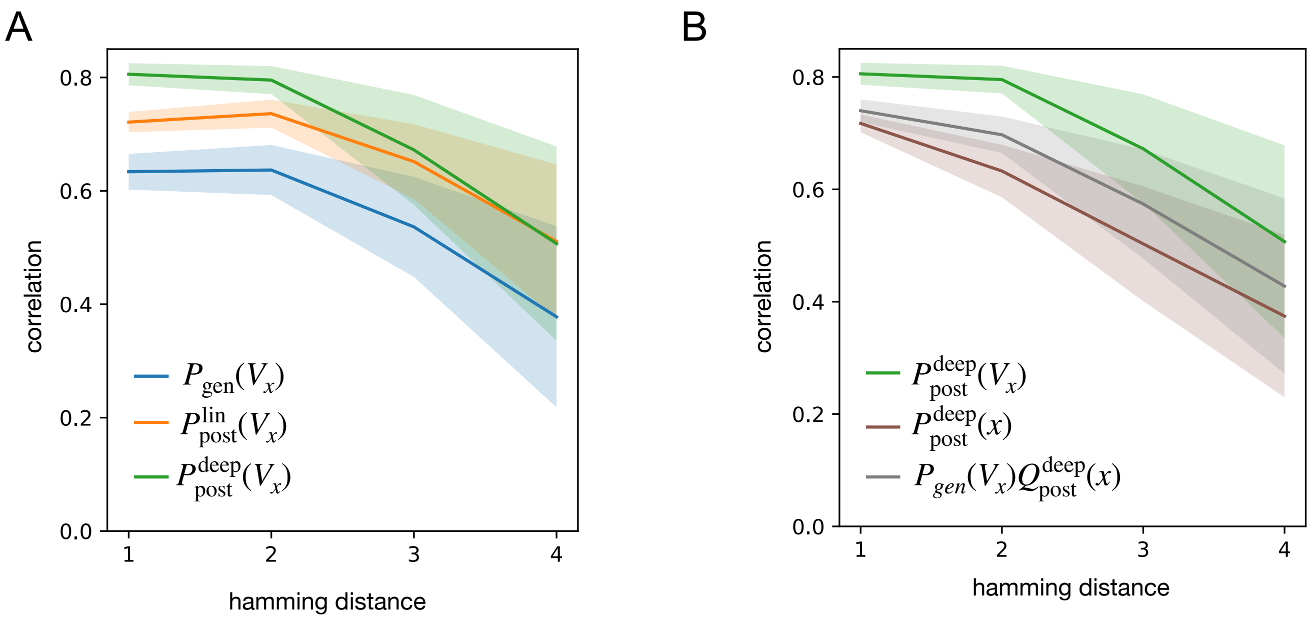

FIG. S4. **Smoothness of $P_{\rm post}$.** **(A)** Comparison of alternative estimators for the number of nearest neighbours defined by different cut-offs in the hamming distance. **(B)** We compare performance for different approximations of $P^{\rm deep}_{\rm post}(V_x)$ which assume smoothness of the whole distribution, $P^{\rm deep}_{\rm post}(x)$, or only of the selection factors, $P_{\rm gen}(V_x)Q^{\rm deep}_{\rm post}(x)$.

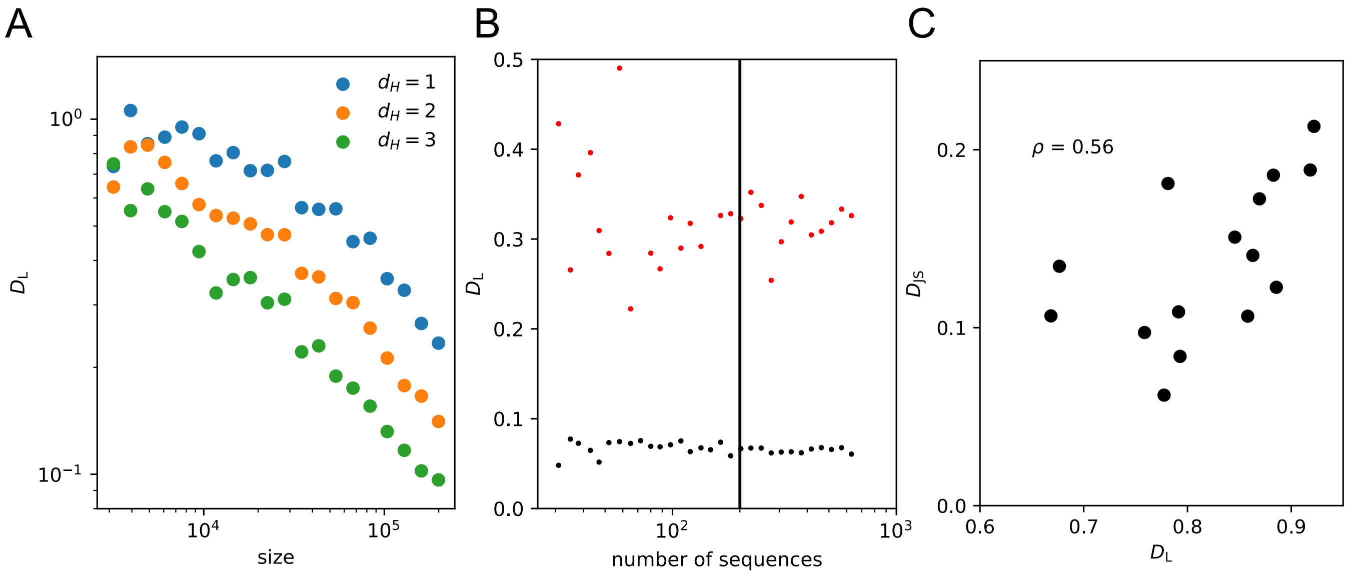

FIG. S5. **Local distance $D_{\rm L}$ between repertoires.** **(A)** We estimate the noise in the model-free version of the estimator $D_{\rm L}$ by comparing subsamples from the same dataset at increasing sizes. We repeat the experiment for different cutoffs in Hamming distance ($d_H \in \{1,2,3\}$) that define the neighbourhood of a sequence. **(B)** Evaluation of the probability for all neighbors of a sequence is computationally intensive. We evaluate the convergence of the model-based version of the estimator $D_{\rm L}$ as a function of how many sequences are evaluated. We observe that for comparisons between similar repertoires (black dots) and very different repertoires (red dots), the estimator converges around 10^2 evaluated sequences. We highlight with a vertical line the sample size (200 sequences) used to produce the results in the main text. **(C)** Replication of the results in Fig. 3A with the model-free version of the estimator for the largest datasets present in our study (six datasets with at least 80,000 unique receptor sequences).

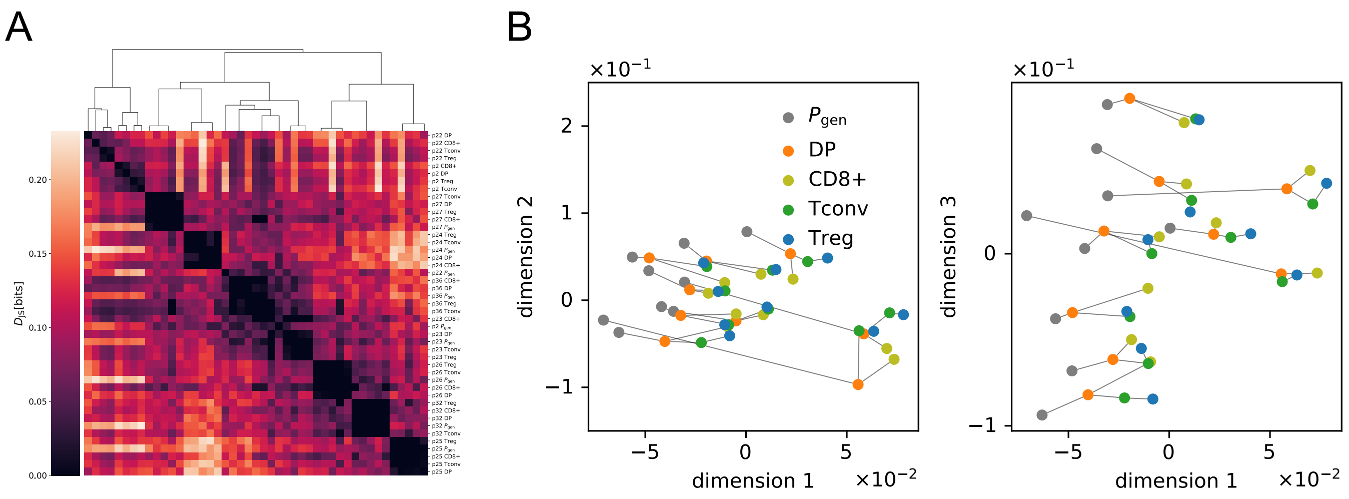

FIG. S6. **Aminoacid Composition Matters.** Replication of Fig. S1A **(A)** and Fig. 1D **(B)** by inferring selection models without aminoacid features. As clearly shown in the distance matrix, the VJ gene features cannot accurately separate intra-individual cell subsets.

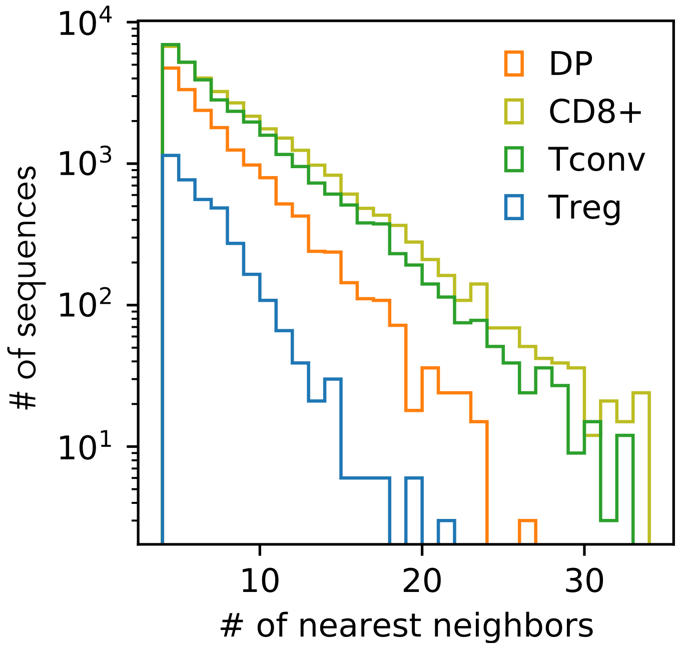

FIG. S7. Histogram of the number of nearest neighbours for each cell type. Due to the smaller dataset size, TCRs in the Treg and DP subset have fewer nearest neighbours on average, resulting in a nosier comparison to the model in Fig. 2E-F.

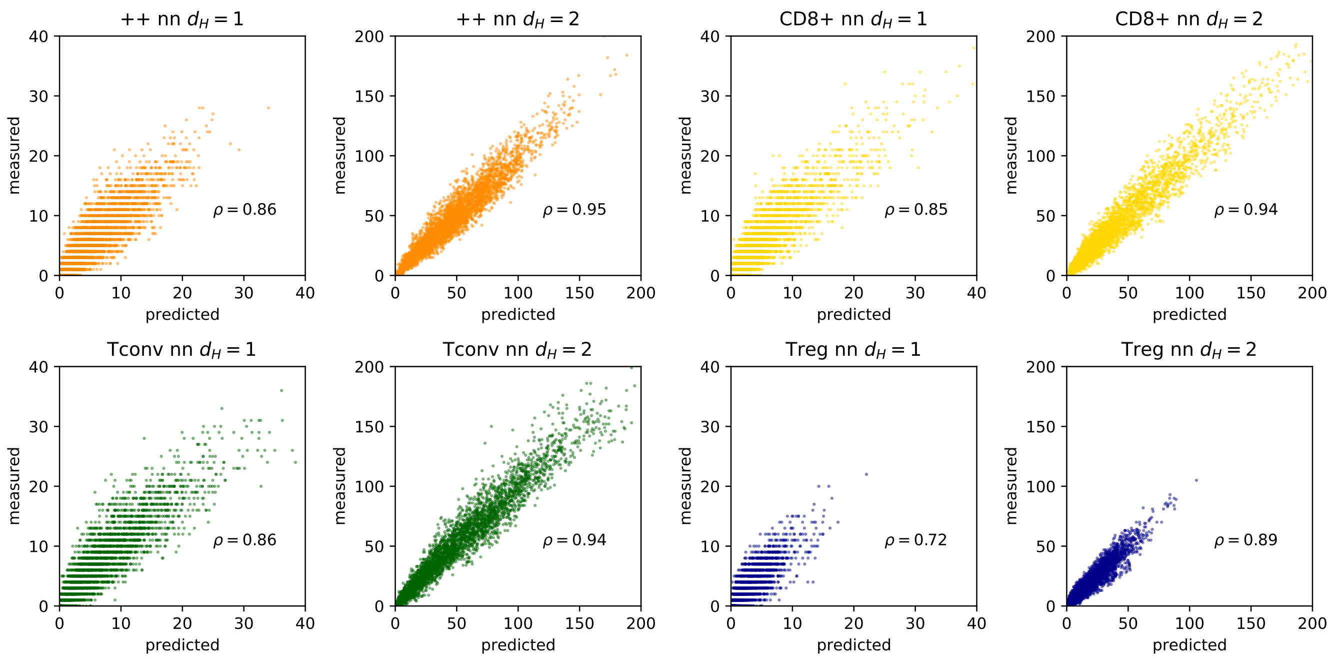

FIG. S8. **Neighborhoods of sequences with high generation probabilities are well predicted by the model**. We sampled $N_0 = 10^6$ sequences using OLGA and kept those that were sampled at least twice and belonged to the 20 most frequent VJ gene groups, resulting in 3899 high generation probability sequences spanning 3 order of magnitudes in P_{post}. The predicted and measured number of neighbours at distance threshold $d_H = 1$ and $d_H = 2$ are shown for the different subsets. Prediction was made using soNNia to which additional calibration for each VJ gene class. The correlation in the prediction is particularly strong and we do not observe significant outliers. This analysis is consistent with a lack of depleted regions around sequences with high generation probabilities.
